# Early neoplastic and metastatic mammary tumours of transgenic mice detected by 5-aminolevulinic acid-stimulated protoporphyrin IX accumulation

**DOI:** 10.1038/sj.bjc.6602840

**Published:** 2005-10-25

**Authors:** A M Dorward, K S Fancher, T M Duffy, W G Beamer, H Walt

**Affiliations:** 1 The Jackson Laboratory, 600 Main St Bar Harbor, ME 04609, USA; 2 Research Division of Gynecology, Department of Obstetrics and Gynecology, University Hospital Zurich, Frauenklinikstrasse 10, CH-8091 Zurich, Switzerland

**Keywords:** photodynamic detection, 5-aminolevulinic acid, protoporphyrin IX, mammary tumour, transgenic mice

## Abstract

A photodynamic technique for human breast cancer detection founded upon the ability of tumour cells to rapidly accumulate the fluorescent product protoporphyrin IX (PpIX) has been applied to transgenic mouse models of mammary tumorigenesis. A major goal of this investigation was to determine whether mouse mammary tumours are reliable models of human disease in terms of PpIX accumulation, for future mechanistic and therapeutic studies. The haeme substrate 5-aminolevulinic acid (5-ALA) (200 mg kg^−1^) was administered to mouse strains that develop mammary tumours of various histological subtypes upon expression of the transgenic oncogenes HRAS, Polyoma Virus middle T antigen, or Simian Virus 40 large T antigen in the mammary gland. Early neoplastic lesions, primary tumours and metastases showed consistent and rapid PpIX accumulation compared to the normal surrounding tissues, as evidenced by red fluorescence (635 nm) when the tumours were directly illuminated with blue light (380–440 nm). Detection of mouse mammary tumours at the stage of *ductal carcinoma* *in situ* by red fluorescence emissions suggests that enhanced PpIX synthesis is a good marker for early tumorigenic processes in the mammary gland. We propose the mouse models provide an ideal experimental system for further investigation of the early diagnostic and therapeutic potential of 5-ALA-stimulated PpIX accumulation in human breast cancer patients.

Synthesis of the fundamental co-factor haeme occurs as a multistep biochemical pathway within the cytoplasmic and mitochondrial compartments of all mammalian cells. Haeme synthesis shows controlled and complex regulation with prominent substrate inhibition at the first step of 5-aminolevulinic acid (5-ALA) production (Ponka, 1999). Exogenous administration of 5-ALA overrides product inhibition, and leads to increased synthesis of the haeme precursors. It is well documented that neoplastic cells derived from multiple human tumour types show an exaggerated capacity for haeme precursor synthesis following 5-ALA exposure relative to normal cells, although the exact contribution to tumour development remains unclear (Collaud *et al*, 2004). Tumours of the skin, bladder, oesophagus, oral cavity, colon, breast, ovary, cervix, leukocytes and brain accumulate the fluorescent haeme precursor protoporphyrin IX (PpIX) following 5-ALA administration (Stummer *et al*, 1998; af Klinteberg *et al*, 1999; Chen *et al*, 2001; Ladner *et al*, 2001; Moesta *et al*, 2001; Betz *et al*, 2002; Bogaards *et al*, 2002; Olivo *et al*, 2003; Stepinac *et al*, 2003; Löning *et al*, 2004). In the case of human colon cancers and their metastases, it was observed that PpIX concentrations were elevated compared to the normal surrounding tissues without exogenous 5-ALA administration (Moesta *et al*, 2001). Investigations to address the mechanism of enhanced PpIX synthesis have focussed on the activity of the haeme synthesis enzymes, and suggest that increased activity of the enzymes involved in porphyrin synthesis – 5-ALA dehydratase and hydroxymethylbilane synthase – combined with reduced activity of ferrochelatase to generate haeme from PpIX, may contribute to the net PpIX accumulation response of tumours (Navone *et al*, 1990; Hinnen *et al*, 1998; Krieg *et al*, 2002). Additional factors that influence PpIX accumulation may relate to the tumour micro-environment (pH, vascularisation) or other properties of tumour cells that could impact upon haeme synthesis, such as altered mitochondrial function, or transport of 5-ALA, porphyrin intermediates, and iron (Collaud *et al*, 2004).

The general property of PpIX loading by tumour cells, either inherently, or following 5-ALA dosing, has stimulated numerous basic research and clinical studies for potential therapeutic and diagnostic uses. PpIX is a natural photosensitiser that generates reactive oxygen intermediates following excitation with 635 nm light. Thus 5-ALA-stimulated PpIX loading has been evaluated for photodynamic therapy (PDT) to eradicate tumour cells (Peng *et al*, 1992; Hua *et al*, 1995; Casas *et al*, 2001; Otake *et al*, 2003). Similarly, PpIX loading stimulated by administration of 5-ALA was investigated for the purpose of photodynamic detection (PDD) of tumour cells (Bedwell *et al*, 1992; Van Hillegersberg *et al*, 1992). This strategy utilises blue light excitation (380–440 nm) to induce red fluorescent emissions (635 nm) from the tumour cells that concentrate PpIX, and has been put to practical use to identify margins of neoplasia during exploratory or tumour de-bulking surgery (Kriegmair *et al*, 1999; Holtl *et al*, 2001; Mayinger *et al*, 2001; Bogaards *et al*, 2005).

5-ALA-stimulated PpIX loading for use in PDD has shown great promise for human breast cancer, both in its application to define primary tumour margins during surgical removal, and for the identification of axillary lymph node metastases; (Ladner *et al*, 2001; Frei *et al*, 2004). We sought to examine the potential for 5-ALA-stimulated PpIX loading in mammary tumours that develop in three well-characterised transgenic models of mammary tumorigenesis. The purpose of this investigation was three-fold: (1) to establish whether transgenic mouse mammary tumours are reliable models of human disease in terms of PpIX accumulation following 5-ALA administration, (2) to determine if the PpIX accumulation response was similar in each oncogenic model of tumorigenesis and (3) to develop an animal model for the study of the cellular and molecular mechanisms of enhanced PpIX accumulation. Our investigation revealed that rapid and significant PpIX loading occurred not only in primary tumours, but also in metastases and very early neoplastic lesions from the different transgenic models. We suggest that ALA-stimulated PpIX loading of early cancerous lesions of human mammary ducts will similarly contribute to early detection of human breast cancer.

## MATERIALS AND METHODS

### Mice

Three transgenic mouse models of mammary tumorigenesis were used for this investigation: (1) Transgenic strain FVB.Cg-Tg(WapHRAS)69Lln Chr Y^SJL^/J (hereafter referred to as HRAS) carries the human RAS oncogene (Harvey rat sarcoma viral oncogene homolog) with mammary tissue-specific gene expression driven by the Whey acidic protein (*Wap*) promoter (Andres *et al*, 1987). The HRAS strain represents a model for male mammary cancer, since solid adenosquamous mammary carcinomas develop only in male transgene carriers, given the integration of this transgene on the Y Chromosome. Care was taken to confirm that developing mammary tumours were not confused with salivary gland tumours that also develop in this strain. (2) The transgenic strain FVB/N-Tg(MMTV-PyVT)634Mul/J, (hereafter referred to as PyVT) carries the Polyoma Virus middle T antigen with mammary tissue-specific expression driven by the mouse mammary tumour virus (MMTV) promoter (Guy *et al*, 1992). Virgin females that carry the transgene develop poorly differentiated, multi-focal, invasive ductal carcinoma by 10–12 weeks of age, with a high incidence of lung metastases stemming from the primary mammary tumour (Lin *et al*, 2003). (3) The transgenic strain C57BL/6J-Tg(WapTAg)1Knw (hereafter referred to as WapTag1) carries the Simian Virus 40 large T antigen (SV40 Tag) with mammary gland-restricted expression under control of the *Wap* promoter (Hüsler *et al*, 1998). Multiparous WapTag1 females develop late-onset mammary adenocarcinomas at 10–13 months of age predominantly of the papillary histological type, but may also show ductal, glandular or solid histology. Additional information about these mammary tumour mouse models can be obtained at the Mouse Tumor Biology Database (http://tumor.informatics.jax.org) via the Mouse Genome Informatics website (www.informatics.jax.org) of The Jackson Laboratory (Näf *et al*, 2002).

All mice were produced and housed in our research colony at The Jackson Laboratory under 14 h : 10 h light : dark cycles. Mice were provided with autoclaved NIH-31 diet (6% fat, 19% protein, vitamin and mineral fortified) (Purina Mills Intl., Brentwood MO) *ad libitum* and HCl-acidified water (pH2.8–3.2) *ad libitum* to retard bacterial growth. Animals were weaned at 18–23 days of age and housed in same sex groups of 3–5 in 51 in^2^ (329 cm^2^) polycarbonate cages containing sterilised White Pine shavings. All animal procedures were approved by the Animal Care and Use Committee of The Jackson Laboratory.

### 5-ALA preparation and administration

5-ALA.HCl (ASAT AG, Zug, Switzerland) was dissolved in sterile saline (0.85% NaCl) immediately prior to administration, and kept in a light-protected vial. All animals were administered a tail vein injection of 5-ALA solution (358 mM) to achieve a final dose of 200 mg kg^−1^ of body weight. Animals were kept in their cages and maintained in a darkened room for a 60–75 min period prior to necropsy.

### Fluorescence detection

Following *in vivo* incubation of 5-ALA, mice were euthanised by cervical dislocation or carbon dioxide asphyxiation. The exteriorised mammary glands were illuminated with blue light (380–440 nm) generated by a D-light system (Storz GmbH, Tuttlingen, Germany) to excite PpIX fluorescence. Digital images were recorded with a Leica DFX 350 camera mounted on a Wild M10 stereo-microscope (Leica Microsystems, Bannockburn, IL, USA) that was fitted with a 635 nm emission filter provided by Storz GmbH. Isolated lungs with mammary tumour metastasis were imaged with a 35 mm camera loaded with 160T slide film (Eastman Kodak, Rochester, NY, USA), that was fitted with the 635 nm emission filter. When fluorescent tumour foci were ⩽1 mm in size, blue background was digitally subtracted from the entire image to provide sufficient contrast for publication purposes (Adobe Photoshop CS, San Jose, CA, USA).

Isolated tumour foci were fixed overnight in Bouin's solution, and histological sections processed for hematoxylin and eosin (H&E) staining. For early neoplastic lesions of WapTag1 mammary glands, a minimum of 30 serial sections were examined to confirm that no mature carcinomas were evident.

### Flow cytometry

Mammary glands or palpable tumour foci from HRAS transgenic males and PyVT transgenic females and littermate controls were excised and minced with fine scissors in phosphate-buffered saline (PBS) containing 2 mg ml^−1^ collagenase type IA (Sigma, St. Louis, MO, USA). Isolated cell suspensions for flow cytometry were generated by a 15-min-incubation in collagenase/PBS under foil cover and disaggregration via successive aspiration through 18 and 20 gauge needles. The cell suspensions were kept on ice, under foil wrap until fluorescence measurements with a FACSVantage SE/DiVa flow cytometer (BD Biosciences, San Jose, CA, USA). PpIX excitation was achieved with a 415.4 nm Innova 90-2 Krypton laser. PpIX fluorescence emissions were recorded following reflection by a 610 nm short pass dichroic mirror to a 590 nm long pass filter (Omega Optical Inc, Brattleboro, VT, USA). Acquistion and analysis of the data for 10 000 tumour cells were performed with FACSDiva ver. 4.0, FACS Convert ver. 1.0 and CellQuestPro ver. 4.0.1 (BD Biosciences, San Jose, CA, USA).

### Statistical analysis

The quantitative fluorescence flow cytometric data, representing a minimum of three independent replicate samples, was analysed by ANOVA with Statview software (SAS Institute Inc, Cary, NC, USA) with *P*<0.05 as the chosen level of statistical significance.

## RESULTS

**Figure 1:**
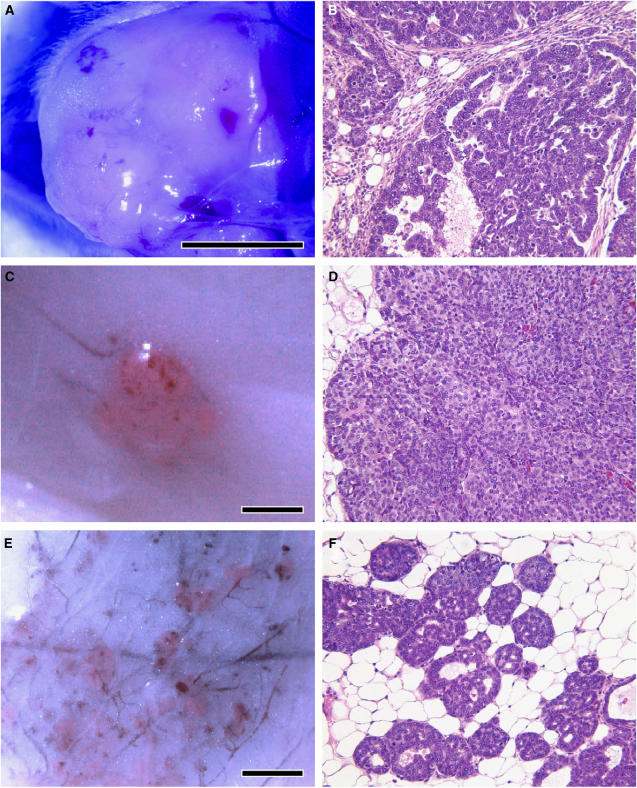
Digital image capture of PpIX-stimulated red fluorescence in tumours of three mammary tumour-bearing transgenic strains 1 h following *in vivo* administration of 200 mg kg^−1^ ALA, with corresponding H&E stained histological sections. (**A**, **B**) Red fluorescence in a papillary adenocarcinoma of a WapTag1 female (77 weeks) (20 ×). (**C**, **D**) HRAS male mammary tumour (11 weeks) showing prominent fluorescence and solid carcinoma histology (20 ×); (**E**, **F**) Fluorescent multifocal tumours of a PyVT female (5 weeks) with solid carcinoma histology (20 ×). Scale bar represents 1 mm.

**Figure 2:**
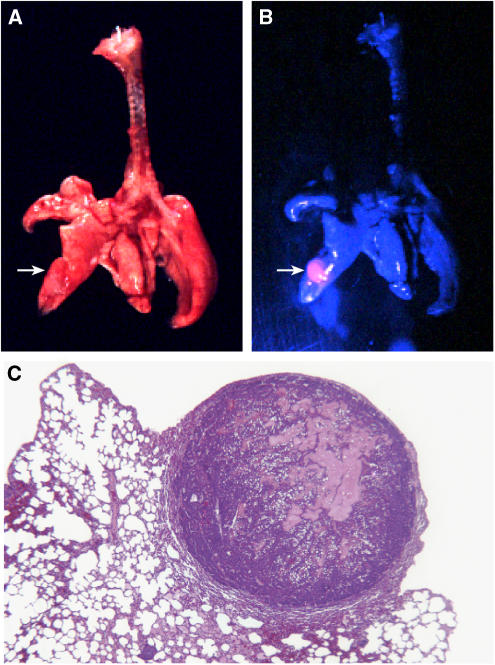
Photographic image of PpIX-stimulated red fluorescence in a metastatic tumour derived from a PyVT strain female (15 weeks) 1 h following 200 mg kg^−1^ ALA administration. (**A**) Brightfield image of isolated bronchial tree. (**B**) Red fluorescence detection of a metastatic tumour in the lung (arrow). (**C**) Representative H&E stained histological section of a tumour metastases from PyVT strain females (4 ×).

Mice bearing palpable mammary tumours were investigated for their PpIX accumulation response following 5-ALA administration. Mice were euthanised and the mammary tumours exteriorised to examine PpIX accumulation under blue light excitation. Mammary tumours in each of the transgenic models responded to 5-ALA administration with significant PpIX accumulation, as measured by tumour-selective red fluorescence relative to the normal surrounding tissues. Fluorescence was most pronounced in PyVT mammary tumours, but was clearly evident in tumours from each transgenic model, despite differences in histological subtype and the gender of the tumour-bearing animal (Table 1, Figure 1). WapTag1 tumours present with the most varied histology, but prominent PpIX fluorescence was observed for tumours of both papillary (Figure 1A and B) and glandular-to-solid subtypes. In HRAS males, tumours 1–2 mm in diameter were easily recognised by red fluorescent emissions following 5-ALA treatment, with similar solid-type histological characteristics as the larger tumour masses (Figure 1C and D). In young PyVT females, the multiplicity of tumour initiation within a single mammary gland was evidenced by red fluorescence following 5-ALA administration, with some of the foci measuring only 0.1 mm (Figure 1E and F). Primary tumours arising in PyVT transgene females frequently metastasize to the lungs. Figure 2 shows an isolated bronchial tree with lung metastases removed from a PyVT strain female. Under blue light, the metastatic focus shines bright red relative to the normal lung tissue, showing that PpIX synthesis remains upregulated in tumour cells with metastatic competence.

**Figure 3:**
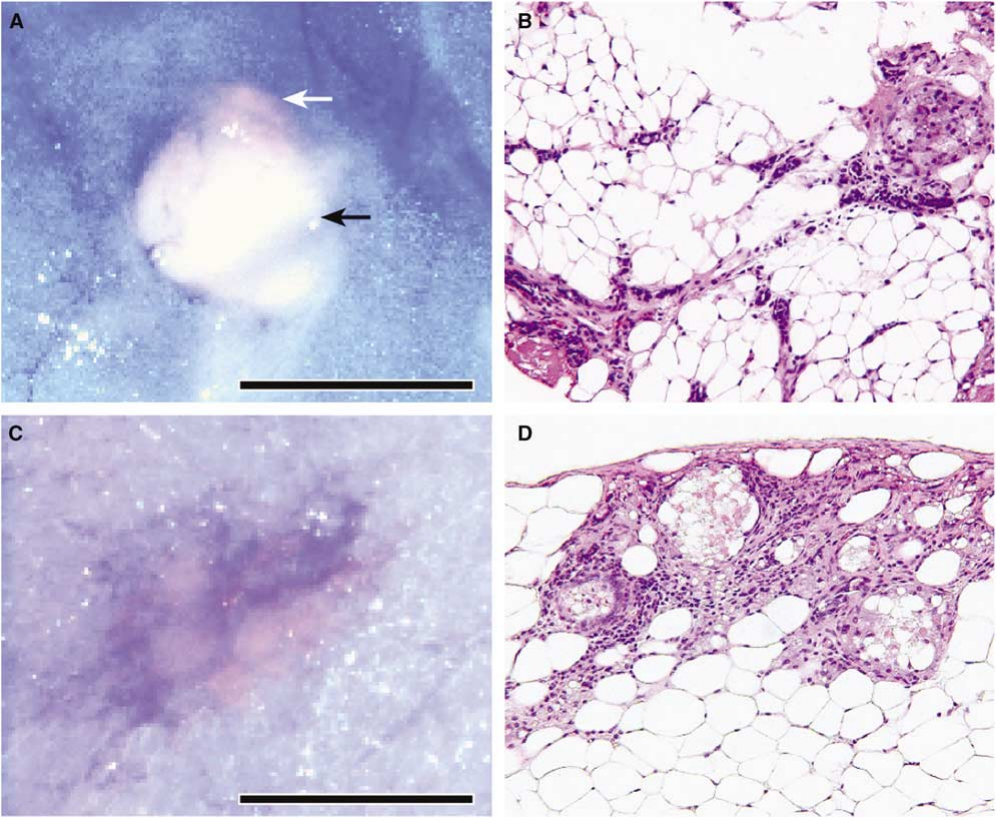
Digital image capture of PpIX-stimulated red fluorescence in early DCIS lesions of two WapTag1 females (41 and 45 weeks) 1 h following *in vivo* administration of 200 mg kg^−1^ ALA. (**A**, **C**) Red fluorescence detection of DCIS in the mammary gland. In (A) The white arrow indicates the region of DCIS, and the black arrow indicates accumulated proteinaceous fluid. (**B**, **D**) Corresponding H&E stained histological sections (20 ×). Scale bar represents 0.5 mm.

Given the evidence for PpIX accumulation in palpable, mature mammary tumours, we administered 5-ALA to WaptTag1 transgenic female mice prior to the anticipated age for palpable tumour presentation, when early stages of tumour progression are predicted for this mouse model. In four independent WapTag1 females, 5-ALA stimulated PpIX accumulation permitted red fluorescence detection of areas histologically confirmed as *ductal carcinoma* *in situ* (DCIS) with accompanying inflammation. Two examples of PpIX stimulated red fluorescence associated with DCIS are illustrated in Figure 3. In Figure 3A, the fluorescent region of DCIS is adjacent to an accumulation of proteinaceous fluid, suggesting the early tumour has stopped the flow of fluids through the duct, while in Figure 3C, the fluorescent region is well demarcated from the surrounding normal gland. Detection of DCIS by PpIX fluorescence indicates that an enhanced capacity to synthesise PpIX is a very early characteristic in the neoplastic transition of these mammary tumours.

**Figure 4:**
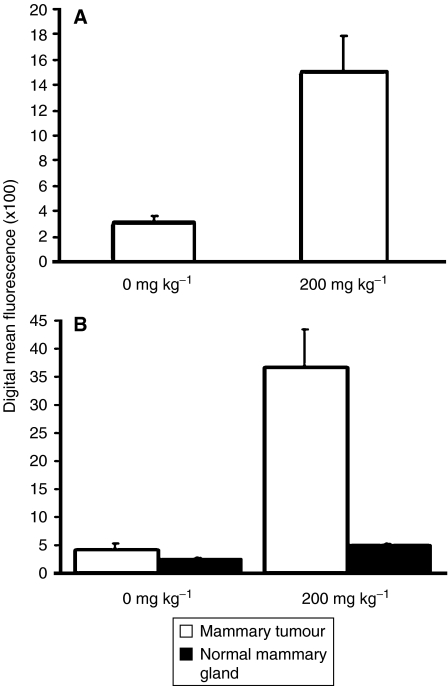
Flow cytometric measurement of digital mean fluorescence in PpIX-loaded tumour cells 1 h following *in vivo* administration of 200 mg kg^−1^ ALA (mean±s.e.). (**A**) Mammary tumour cells from HRAS males. (**B**) Normal mammary gland and mammary tumour cells from PyVT females.

PpIX-stimulated fluorescence was quantified by flow cytometric analysis in palpable (5–10 mm diameter) homogeneous mammary tumours of HRAS transgenic males and PyVT transgenic females. A comparison of the fluorescence emitted from isolated HRAS tumour cells derived from 5-ALA-primed *vs* control tumour-bearing males is shown in Figure 4A: the HRAS tumours exposed to 5-ALA had a significant, five-fold increase in fluorescence emissions. Similarly, the mammary tumour cells isolated from PyVT strain females primed with 5-ALA showed a significant, seven-fold increase in fluorescence emissions compared to tumour cells obtained from control animals (Figure 4B). To illustrate the significant difference in the 5-ALA response of mammary tumors *vs* the normal surrounding tissues, we also compared the PpIX fluoresence emissions of normal mammary glands obtained from nontransgene carrier, virgin littermates of the PyVT females. Like the tumour cells, the normal mammary glands were either exposed to 5-ALA (200 mg kg^−1^) for 1 h *in vivo* or were obtained directly from unprimed control females. 5-ALA administration induced a significant, two-fold increase in red fluorescence emissions in the normal mammary gland, although this increase was significantly less than the seven-fold increase observed in 5-ALA-primed PyVT tumour cells, and did not interfere with the visual margins of differential red fluorescence observed between tumour cells and normal tissues in the intact animal (Figure 1E).

## DISCUSSION

To establish whether transgenic mouse models of mammary tumorigenesis are relevant models for human breast cancer in terms of their response to 5-ALA, we have evaluated three well-characterised transgenic strains that develop mammary tumours under powerful oncogenic stimulation – HRAS, PyVT and SV40 Tag – for their ability to accumulate PpIX following administration of 5-ALA. Our findings showed that the mammary tumours from each transgenic strain rapidly and selectively converted 5-ALA to PpIX, and showed five- to seven-fold enhanced fluorescence compared with surrounding normal tissues under blue light excitation. Similar to the primary mammary tumours, spontaneous metastatic lesions to the lungs of tumour bearing PyVT mice were readily demarcated by PpIX accumulation and red fluorescence under blue light, suggesting that the cellular mechanisms responsible for PpIX accumulation are retained within tumour populations that acquire metastatic competence. This follows the findings of Frei *et al* (2004) who found that metastatic breast cancer cells in axillary lymph nodes of human patients could be identified by PpIX accumulation.

PpIX accumulation in the primary tumours of each strain was consistent, despite fundamental differences in histological subtype, latency of onset, metastatic potential and gender of the tumour-bearing host. Thus, the transgenic models parallel the findings for human breast cancer cases in that the PpIX accumulation phenotype is not selective for any particular tumour histological subtype (Ladner *et al*, 2001). Most significantly, our observations of PpIX accumulation in early DCIS of the WapTag1 transgenic mammary glands also suggested this phenotypic switch is an early event in the tumorigenic process. If enhanced PpIX synthesis can similarly be utilized as an early marker for human breast cancer, the impetus is strong to develop noninvasive imaging strategies that take advantage of tumour cell-selective changes in PpIX biochemistry for early detection.

A new avenue of clinical tumour imaging for human breast cancer has opened with the availability of fiberoptic ductoscopy (Mokbel *et al*, 2005; Sauter *et al*, 2005). Endoscopes of fine diameter (<1 mm) inserted through the breast nipple can gather three-dimensional structural information of the mammary ducts, and are under investigation as a tool for breast cancer detection. The high quality of optical imaging allows localisation of micro-pathology, such as DCIS of the epithelial lining within the mammary ducts. Our findings for enhanced PpIX accumulation in early DCIS of mouse mammary tumours suggests that fiberoptic ductoscopy combined with a fluorescence detection system for PpIX could increase the specificity and sensitivity of ductoscopy for early tumour detection in the human breast.

Confirmation that mouse models of both male and female mammary tumorigenesis parallel the clinical findings for human breast cancer in terms of PpIX synthesis could lead to new lines of investigation for the detection, diagnosis and therapy of breast cancer. One advantage of the mouse models of mammary tumorigenesis over cell culture models is that cellular events leading to the PpIX accumulation phenotype can be genetically and chronologically mapped according to the various stages of tumorigenesis. Furthermore, an investigation as to whether altered regulation of the haeme synthesis pathway is important for tumour development can be further addressed in genetically engineered mouse models of mammary tumorigenesis.

## Figures and Tables

**Table 1:** Group size and histological characteristics of transgenic strain mammary tumours examined for PpIX accumulation

**Strain**	**Gender (M/F)**	**Group size (*n*)**	**Histological tumour type**
HRAS	M	20	Solid adenocarcinoma
PyVT	F	20	Multifocal, solid adenocarcinoma
WapTag1	F	8	Papillary adenocarcinoma, glandular/solid adenocarcinoma
